# Peripheral airways type 2 inflammation, neutrophilia and microbial dysbiosis in severe asthma

**DOI:** 10.1111/all.14732

**Published:** 2021-01-26

**Authors:** Adnan Azim, Ben Green, Laurie Lau, Hitasha Rupani, Nivenka Jayasekera, Kenneth Bruce, Peter Howarth

**Affiliations:** ^1^ Faculty of Medicine Academic Unit of Clinical and Experimental Sciences University of Southampton Southampton UK; ^2^ NIHR Respiratory Biomedical Research Unit University Hospital Southampton Southampton UK; ^3^ Portsmouth Hospitals NHS Trust Queen Alexandra Hospital London UK; ^4^ Newham University Hospital Barts Health Trust London UK; ^5^ Molecular Microbiology Research Laboratory Pharmaceutical Science Division King's College London London UK

**Keywords:** 16srRNA, IL‐13, neutrophils, peripheral airways, severe asthma

## Abstract

**Background:**

IL‐13 is considered an archetypal T2 cytokine central to the clinical disease expression of asthma. The IL‐13 response genes, which are upregulated in central airway bronchial epithelial of asthma patients, can be normalized by high‐dose inhaled steroid therapy in severe asthma. However, this is not the case within the peripheral airways. We have sought to further understand IL‐13 in the peripheral airways in severe asthma through bronchoalveolar analysis.

**Methods:**

Bronchoalveolar lavage samples were collected from 203 asthmatic and healthy volunteers, including 78 with severe asthma. Inflammatory mediators were measured using a multiple cytokine immunoassay platform. This analysis was replicated in a further 59 volunteers, in whom 16S rRNA analysis of BAL samples was undertaken by terminal restriction fragment length polymorphism.

**Results:**

Severe asthma patients with high BAL IL‐13, despite treatment with high‐dose inhaled corticosteroids, had more severe lung function and significantly higher BAL neutrophil percentages, but not BAL eosinophils than those with normal BAL‐13 concentrations. This finding was replicated in the second cohort, which further associated BAL IL‐13 and neutrophilia with a greater abundance of potentially pathogenic bacteria in the peripheral airways.

**Conclusion:**

Our findings demonstrate a steroid unresponsive source of IL‐13 that is associated with BAL neutrophilia and bacterial dysbiosis in severe asthma. Our findings highlight the biological complexity of severe asthma and the importance of a greater understanding of the innate and adaptive immune responses in the peripheral airways in this disease.

## Graphical abstract

In this study, we stratified biologic naïve severe asthma patients treated with high‐dose inhaled corticosteroid therapy by their bronchoalveolar lavage IL‐13. Patients with high bronchoalveolar lavage IL‐13, despite steroid therapy, have a higher percentage of bronchoalveolar lavage neutrophils. We replicated these findings in a second smaller cohort, which also associated high bronchoalveolar lavage IL‐13 with bacterial dysbiosis.

Abbreviations: BAL, bronchoalveolar lavage; ICS, inhaled corticosteroids; OCS, oral corticosteroids.

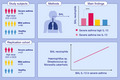

**Abbreviations**
ACQ6: 6 item asthma control questionnaire
BAL: bronchoalveolar lavage
BD: bronchodilator
BDPe: beclomethasone diproprionate equivalent
CLCA1: chloride channel, calcium‐activated family member 1
FEV_1_
: forced expiratory volume in 1 second
HC: healthy control
IL: Interleukin
IQR: interquartile range
MA: mild asthma
POSTN: periostin
PPM: potentially pathogenic micro‐organisms
SA: severe asthma
SERPINB2: serine peptidase inhibitor clade B member 2
T‐RFLP: terminal restriction fragment length polymorphism

## 1 INTRODUCTION

Asthma is a highly prevalent airway disease that affects more than 300 million people across the world.^1^ In the majority of patients, low‐dose inhaled corticosteroid therapies are able to reduce airway inflammation and successfully control symptoms and exacerbations. However, approximately 10% of patients with asthma continue to struggle, despite treatment with high‐dosage inhaled corticosteroids (ICS) plus a second controller and/or systemic corticosteroids ^2^: these patients with persistent uncontrolled asthma suffer from high rates of morbidity ^3^ and account for a disproportionate use of asthma healthcare resources.^4^

In this difficult to control asthma population, the phenotypic heterogeneity ^5^, ^6^ and varied response to currently available treatments ^7^, ^8^, ^9^ testify to the complexity and variability in the underlying disease‐related biology. The pleiotropic cytokine interleukin‐13 (IL‐13) is increased in bronchoalveolar lavage fluid and overexpressed in sputum and bronchial biopsy specimens of severe asthma patients.^10^ Produced by type‐2 helper T‐cells (Th2), mast cells, basophils and ILC2 cells, IL‐13 is considered an archetypal T2 cytokine central to asthma pathophysiology. The IL‐13 response genes, CLCA1 (chloride channel, calcium‐activated family member (a), POSTN (periostin) and SERPINB2 (serine peptidase inhibitor clade B member (b) have been shown to be upregulated in central airway bronchial epithelial cells in non‐steroid treated asthma ^11^ and normalized in the central airways by high‐dose inhaled steroid therapy in those with severe asthma.^12^ However, this is not the case within the peripheral airways,^12^ which represent the majority of airway luminal surface area, and are increasingly appreciated to contribute to the clinical expression of severe asthma.^13^ We have thus sought to gain insight into persistent IL‐13 activity in the peripheral airways of severe asthma through bronchoalveolar lavage analysis.

## 2 METHODS

### 2.1 Study populations

Bronchoalveolar lavage samples were collected from a large cohort of patients and findings validated in a second smaller cohort. All patients provided informed consent to the study, which was conducted in accordance with the Helsinki Declaration. Independent ethics committee approval was obtained (MREC No. 05/Q1702/165). All severe asthma participants were biologic therapy naïve but fulfilled the following severity criteria: maintenance treatment with high‐dose inhaled steroids plus at least 2 add on maintenance therapies (including long‐acting beta‐agonists, leukotriene receptor antagonists or oral steroids) and treatment at GINA/British Thoracic Society asthma management steps 4 or 5. Detailed clinico‐physiological characterization was undertaken. There were two comparator groups. Healthy volunteers, all of whom had no history of lower airways disease, had normal bronchial reactivity on histamine inhalation challenge and were on no respiratory medications. Those labelled mild asthma had a diagnosis of asthma, had abnormal airway hyperreactivity and were either not receiving any maintenance asthma therapy or were managed with low‐dose inhaled corticosteroids (GINA step 1 and 2).

### 2.2 Bronchoscopy and BAL processing

Flexible bronchoscopy was performed as described in concordance with established guidelines.^14^ Bronchoalveolar lavage (BAL) was undertaken by instilling six sequential 20‐mL aliquots of pre‐warmed (37°C) normal saline into a subsegmental bronchus of the right upper lobe followed by gentle suction 10 seconds after each instillation. BAL fluid was filtered (BD Falcon Cell Strainer) and then centrifuged at 800 g for 10 minutes at 4°C. Cell pellets were resuspended in phosphate‐buffered saline for cytospins, and the supernatant was stored at –80°C for later analysis. Cells were stained by using a rapid Romanowsky stain (Raymond Lamb Ltd) to distinguish between macrophages, neutrophils and eosinophils, and 400 cells were counted blind by using coded samples. BAL supernatant concentrations of various cytokines were measured by electrochemiluminescence immunoassay.

### 2.3 Inflammatory cell cut‐offs

A 2% cut‐off was used for eosinophilic and non‐eosinohilic; 7.5% for neutrophilic and non‐neutrophilic. These were used to define four inflammatory phenotypes: eosinophilic, neutrophilic, mixed granular and paucigranular.

### 2.4 Cytokine analysis

Inflammatory mediators were measured using a V‐plex multiple cytokine immunoassay platform (Meso Scale Discovery, MSD) as per the manufacturer's instructions. The assay use SUFO‐Tag labelled Detection Antibody for electroemiluminescence.

### 2.5 Microbial analysis

16S rRNA analysis of BAL samples was undertaken by T‐RFLP as described previously.^15^ Briefly, nucleic acid was extracted directly from BAL samples and a 927 base fragment of the 16S rRNA genes amplified with a 5′ IRD700‐tagged primer. Subsequently, the amplified 16S rRNA genes were digested with CfoI and resolved on a IR2 automated DNA sequencer (LI‐COR Biosciences). T‐RFLP profiles were analysed using Phoretix one‐dimensional advanced software, v.5.10 (Nonlinear Dynamics). T‐RF band sizes were determined by comparison with MicroSTEP‐15a (700 nm) size marker (Microzone). T‐RF band volume was determined and expressed as a percentage of the total volume of bands detected in each electrophoretic profile.

### 2.6 Statistical analysis

Due to the non‐normal distribution of data, descriptive variables are presented as median (interquartile range) and binary variables as prevalence (%). A Kruskal‐Wallis or chi‐square test was used for between group comparisons of continuous and categorical data, respectively. Correlations were assessed using Spearman's rank tests. All tests were two‐tailed, and a *p*‐value <0.05 was considered significant. Dunn's method was used to correct for multiple comparisons using statistical hypothesis testing. Statistical analysis was performed with the SPSS 26.0 software and Graphpad Prism 8. Severe asthma patients were stratified into tertiles, based on their BAL IL‐13 concentrations, to facilitate an extreme group analysis.

## 3 RESULTS

### 3.1 Clinical characteristics of the bronchoscopy patient cohort

Bronchoalveolar lavage (BAL) samples were obtained at flexible bronchoscopy from 203 volunteers; 45 non‐asthmatic healthy volunteers, 80 non‐steroid treated asthmatics and 78 asthmatics with severe disease (Table 1). Compared to patients with mild asthma, patients with severe asthma were older, less atopic, more likely to have a smoking history and have poorer lung function despite high‐dose inhaled corticosteroid therapy (Table 1). Pairwise comparisons between groups, corrected by Dunn's test for multiple comparisons, demonstrate mild asthma (*p* < 0.001) and severe asthma (*p* = 0.004) to have higher BAL eosinophils than healthy controls; there was no statistically significant difference between Mild and severe asthma. In contrast, there was no statistically significant difference in BAL neutrophils between mild asthma and healthy controls, but these were higher in severe asthma compared to mild asthma (*p* < 0.001) and healthy controls (*p* < 0.001).

**TABLE 1:** Clinical characteristics of bronchoscopy cohort

	Healthy volunteers *(45)*	Mild asthma *(80)*	Severe asthma *(78)*	*p* value
Age	23.00 (9.50)	23.5 (14.19)	45.0 (26.0)	<0.001
Gender (% female)	55.6%	76.4%	69.2%	Ns
Atopic (% positive)	24.4%	93.8%	67.9%	<0.001
BMI	24.22 (5.39)	24.00 (4.26)	31.84 (13.12)	<0.001
Smoking (% never)	86.7%	90.0%	66.7%	0.001
Post BD FEV_1_% pred	104.76 (15.71)	108.99 (10.71)	74.07 (25.31)	<0.001
Post BD FEV_1_/FVC	86.80 (10.50)	81.10 (11.65)	73.65 (20.98)	<0.001
BDPe	n/a	0 (400)	2390 (1000)	<0.001
Maintenance prednisolone	n/a	n/a	50%	n/a
ACQ6	n/a	0.86 (1.29)	3.29 (0.99)	<0.001
BAL Eosinophil %	0.25 (0.50)	1.00 (2.20)	0.50 (1.41)	<0.001
BAL Neutrophil %	2.30 (3.65)	2.00 (2.44)	6.00 (12.63)	<0.001

Quantitative variables expressed as median (interquartile range), between group comparisons by Kruskal‐Wallis test. Categorical variables expressed as percentages within each group, between group comparisons by chi‐Square test. Maintenance prednisolone defined as minimum of 5 mg/day.

Abbreviations: ACQ6, 6 item asthma control questionnaire; BDPe, beclomethasone diproprionate equivalent; BD, bronchodilator; BAL, bronchoalveolar lavage; BMI, body mass index; FEV1, forces expiratory volume in 1 second; FVC, forced vital capacity.

**FIGURE 1:**
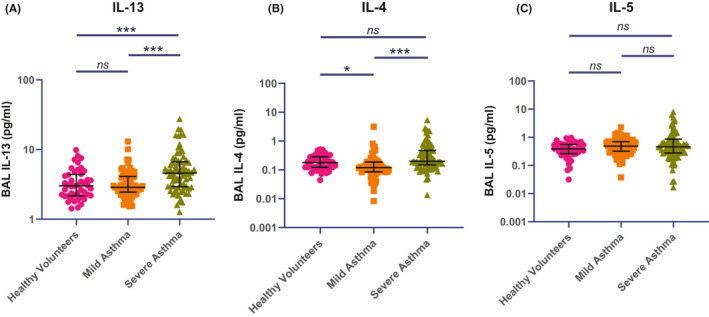
Type 2 cytokine concentrations in healthy volunteers, mild asthma and severe asthma patients. Between group comparisons by Kruskal‐Wallis with pairwise comparisons corrected for multiple comparisons by Dunn's Method. (A) Bronchoalveolar Lavage Interleukin 13, (B) Bronchoalveolar Lavage Interleukin 4, (C) Bronchoalveolar Lavage Interleukin 5. Between group comparison by Kruskal‐Wallis test, ***p < 0.001

There was no difference in median (IQR) BAL IL‐13 concentrations (pg/ml) between mild asthma, 2.87 (1.66), and Health Controls, 3.02 (2.23); but BAL IL‐13 was increased in severe asthma, 4.60 (3.74) (Figure 1). Other classically recognized T2 cytokines, IL‐4 and IL‐5, were not significantly elevated in the BAL of severe asthma patients compared to healthy controls (Figure 1, Table S1). In patients with severe asthma, there is strong association, measured by Spearman rank correlation, between BAL IL‐13 and IL‐ 4 concentrations, *r* = 0.749, *p* < 0.001 but weaker association between BAL IL‐13 and IL‐5 concentrations, 0.410, *p* < 0001.

### 3.2 Extreme group analysis of severe asthma BAL IL‐13

**FIGURE 2:**
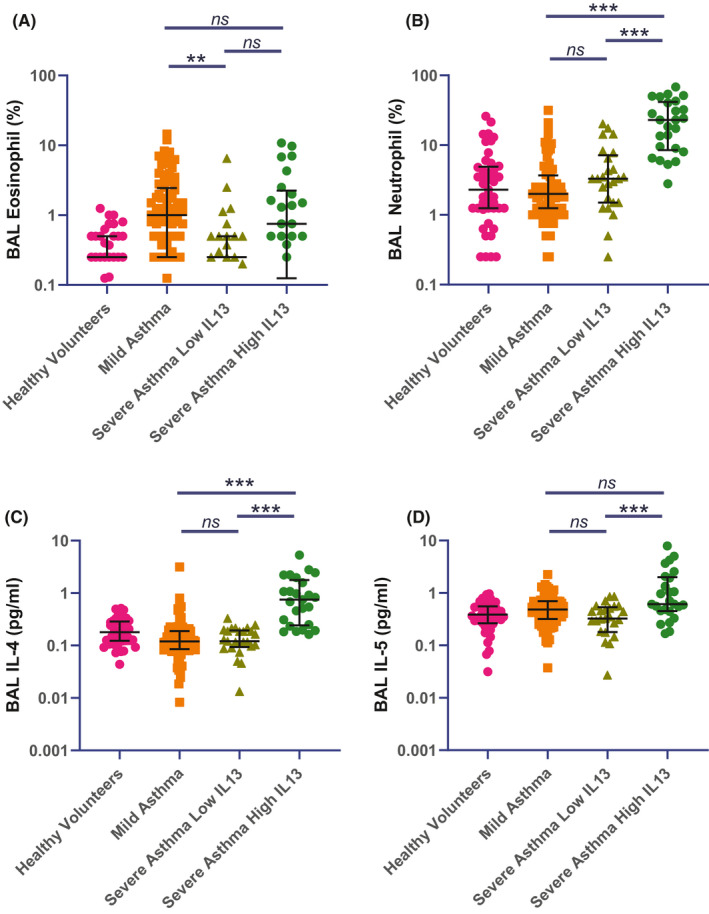
Bronchoalveolar Inflammatory Cell counts and Type 2 cytokine concentrations in healthy volunteers, mild asthma and severe asthma patients from the top and bottom tertile of patients ranked by Interleukin 13 concentrations. Between group comparisons by Kruskal‐Wallis with pairwise comparisons corrected for multiple comparisons by Dunn's Method. (A) Bronchoalveolar Lavage Eosinophil Percentages, (B) Bronchoalveolar Lavage Neutrophil Percentages, (C) Bronchoalveolar Lavage Interleukin 4, (D) Bronchoalveolar Lavage Interleukin 5. Between group comparison by Kruskal‐Wallis test, **p <0.01, ***p < 0.001

In the BAL IL‐13 tertiles, severe asthma patients with high BAL IL‐13 and those with low BAL IL‐13 were similar in terms of demographics and were treated with similar doses of inhaled corticosteroids (Table 2). However, high BAL IL‐13 patients were less often atopic and had poorer lung function. They were also distinct in term of inflammatory cell counts: high BAL IL‐13 patients showed no difference in terms of BAL eosinophils but did have increased BAL neutrophils (Figure 2 and Table S2). In patients with severe asthma, IL‐13 correlated, by Spearman rank, moderately with BAL neutrophilia, 0.580, *p* < 0.001 but only very weakly with BAL Eosinophilia, 0.271, *p* = 0.017. BAL IL‐4 and BAL IL‐5 were both increased in the high BAL IL‐13 group compared with the low IL‐13 severe asthma group.

**TABLE 2:** Characteristics top and bottom tertile of patients ranked by bronchoalveolar lavage Interleukin 13 concentrations

	Severe asthma low tertile IL13 *(25)*	Severe asthma high tertile IL13 *(25)*	*p* value
BAL IL−13	2.47 (0.71)	8.81 (9.13)	<0.001
Age	42.00 (25.00)	49.00 (24.00)	Ns
Gender (% female)	84%	60%	Ns
Atopic (% positive)	76%	48%	0.040
BMI	32.79 (16.75)	29.48 (12.03)	Ns
Smoking (% never)	76%	72%	Ns
Post BD FEV_1_% pred	85.13 (32.50)	68.10 (34.42)	0.037
Post BD FEV1/FVC	78.89 (17.94)	66.45 (23.63)	0.012
BDPe	2400 (1310)	2400 (1300)	Ns
Maintenance prednisolone	44.0%	56.0%	Ns
ACQ6	3.57 (1.98)	3.29 (0.71)	Ns
BAL IL−4	0.12 (0.10)	0.75 (1.53)	<0.001
BAL IL−5	0.32 (0.35)	0.61 (1.54)	0.001

Quantitative variables expressed as median (interquartile range), between group comparisons by Mann‐Whitney U test. Categorical variables expressed as percentages within each group, between group comparisons by chi‐Square test. Maintenance prednisolone defined as minimum of 5 mg/day.

Abbreviations: ACQ6, 6 item asthma control questionnaire; BAL, bronchoalveolar lavage; BD, bronchodilator; BDPe, beclomethasone diproprionate equivalent; BMI, body mass index; FEV1, forces expiratory volume in 1 second; FVC, forced vital capacity; IL, interleukin.

### 3.3 Replication of bronchoscopy findings

Bronchoalveolar lavage (BAL) samples were obtained in a second smaller study from 59 volunteers: 7 non‐asthmatic healthy volunteers, 25 non steroid treated asthmatics and 27 asthmatics with severe disease. Extreme group analysis of the severe asthma patients, defined by BAL IL‐13 tertiles, once again demonstrate worse disease in high BAL IL‐13 patients with worse lung function. In this smaller cohort, they also had worse self‐report asthma control and required higher doses of inhaled corticosteroid (Table 3). We were able to replicate our finding that there are no differences in BAL eosinophils between these groups but an increase in BAL neutrophils (Table 3 and Figure S1).

**TABLE 3:** Characteristics of healthy volunteers, mild asthma and top and bottom tertiles of patients ranked by Bronchoalveolar Lavage Interleukin 13 concentrations from the Replication Cohort

	Healthy volunteers *(7)*	Mild asthma *(25)*	Severe asthma low IL13 *(9)*	Severe asthma High IL13 *(9)*	*p* value
Age	27.00 (15.00)	24.00 (8.00)	42.00 (23.00)	51.00 (21.00)	0.001
Gender (% female)	57.1%	72.0%	66.7%	77.8%	Ns
Atopic (% positive)	85.7%	96.0%	55.7%	77.8%	Ns
Smoking (% never)	100%	91.7%	55.6%	55.6%	0.016
PostBD FEV_1_%pred	108.46 (11.26)	104.58 (16.49)	88.98 (33.53)	68.87 (48.53)	<0.001
BDPe	n/a	n/a	1600 (940)	2000 (1900)	Ns
Maintenance Prednisolone	n/a	n/a	66.7%	33.3%	Ns
ACQ6	n/a	1.00 (1.00)	2.71 (1.00)	3.43 (2.00)	<0.001
BAL IL−13	2.16 (1.40)	1.39 (1.50)	2.27 (0.82)	9.67 (4.22)	<0.001
BAL Eosinophil %	0.0 (0.80)	1.80 (3.45)	0.50 (1.65)	1.30 (4.53)	0.008
BAL Neutrophil %	2.00 (9.20)	3.00 (5.70)	5.80 (11.15)	36.50 (48.75)	<0.001

Quantitative variables expressed as median (interquartile range), between group comparisons by Mann‐Whitney U test. Categorical variables expressed as percentages within each group, between group comparisons by ch‐ Square test.

Abbreviations: ACQ6, 6 item asthma control questionnaire; BAL, bronchoalveolar lavage; BD, bronchodilator; BDPe, beclomethasone diproprionate equivalent; FEV1, forces expiratory volume in 1 s; IL, interleukin.

### 3.4 Microbial analysis

**FIGURE 3:**
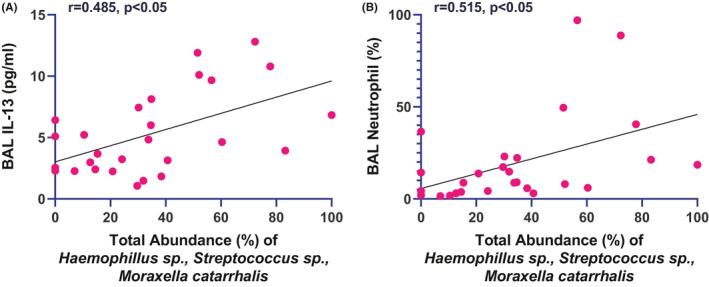
Correlation between total abundance of potentially pathogenic bacteria and Bronchoalveolar Lavage concentrations in the replication cohort. (A) Interleukin 13, (B) Neutrophils (%)

Data were available from the second cohort of BAL microbial analysis by T‐RFLP. In patients with severe asthma, colonization with potentially pathogenic bacteria, as represented by combined abundance of *Moraxella catarrhalis*, *Haemophilus sp* and *Streptococcus* sp, correlated with BAL neutrophil percentage and BAL IL‐13 (Figure 3).

## 4 DISCUSSION

We have extended our bronchial brush findings of persistent IL‐13 gene expression in the peripheral airways of patients with severe asthma,^12^ by demonstrating elevated levels of BAL IL‐13 in patients with severe asthma compared to healthy volunteers and those with mild asthma. Interleukin‐13 is commonly associated with T2 disease processes that are linked to eosinophilia and are corticosteroid sensitive ^10^, ^16^; accordingly, it might be anticipated that both BAL eosinophils and BAL IL‐13 would be suppressed by the high dose inhaled corticosteroids therapy used in these patients. Our finding demonstrates this to be true for some severe asthma patients, identified as the low BAL IL‐13 group. However, we also identify a number of severe asthma patients, who despite, their therapy have elevated BAL IL‐13. These patients can be distinguished from healthy controls, mild asthmatics and severe asthmatics with low BAL IL‐13 concentrations, by having particularly severe lung function and airway neutrophilia. We have replicated these findings in a second cohort, which further associates BAL IL‐13 and BAL neutrophils to the abundance of potentially pathogenic microbes.

Though arguably an oversimplistic paradigm,^17^ IL‐13 is typically considered to be reciprocally regulated ^18^ to one of the central mediators for airway neutrophil recruitment, IL17A.^19^ Neutrophils are usually the first non‐resident immune cells to arrive at a site of inflammation ^20^ but one of the functions of the T2 cytokine milieu is to cause an IL‐4R mediated inhibition of neutrophil activity.^21^, ^22^, ^23^ In helminth infections, failure of this regulatory T2 response leads to IL‐17 mediated lung tissue damage.^24^ Division of the subjects into inflammatory phenotypes (Table S3) identified higher IL‐13 BAL levels in both neutrophilic and mixed inflammatory phenotypes. The parallel increase of BAL IL‐13 and BAL neutrophilia in the high tertile population could all represent a mixed inflammatory phenotype, as IL‐5 concentrations were higher in this sub‐group than in the low IL‐13 tertile. Arguing against such a consideration was the finding that eosinophil proportions (and numbers –Table S2) were no different from the low IL‐13 tertile, which would be the standard way to define inflammatory phenotypes. However, we have previously identified that ECP levels in BAL are higher in severe asthma than mild asthma despite BAL eosinophils being no different,^14^ so eosinophil numbers alone do not reflect activity and may overlook completely degranulated cells. As such, this possibility cannot be entirely excluded but the data is more consistent with the peripheral airway IL‐13 being linked to neutrophilic disease.

In considering the source of BAL IL‐13 in these patients, our previous epithelial brushing study identified peripheral airway epithelial cells responding to IL‐13 but not generating it.^12^ Rather, as the severe asthma patients were all treated with high dose inhaled steroid therapies, the origin of observed IL‐13 must be steroid unresponsive, making eosinophils and Th2 cells unlikely. Recent attention has focused on ILC2 cells ^25^ as a possible source, however, in addition to the weak relationship between IL‐13 and IL‐5, this cell population is generally not recognized to generate IL‐4, as was seen in our analysis. An alternative source of IL‐4 and IL‐13 are mast cells, a cell population increased within the peripheral airways of severe asthma and whose activation is poorly supressed by steroid therapy.^12^, ^26^

Traditionally, mast cells are linked to allergy but are also recognized to be important in innate immune responses and can be activated by bacterial products.^27^ Airway colonization by potentially pathogenic bacteria is associated with airway neutrophilia ^28^ and corticosteroid‐resistance ^29^; consistent with our observations in this study with BAL IL‐13. It is unclear, through this cross‐sectional study design, whether bacteria are triggers for IL‐13 production, if the elevated IL‐13 levels alter the environment in favour of these pathogens or if these represent two separate co‐incidental events. The replication of the findings makes the co‐incidental consideration less plausible. Moreover, where therapeutic strategies have broadly been unsuccessful for neutrophilic asthma,^19^ it is notable that azithromycin, which leads to exacerbation reduction for eosinophilic and non‐eosinophilic patients,^30^ was most effective in those patients colonised by H. influenzae.^31^ There is a wealth of data supporting the observation that human microbiota influences the maturation and function of the host immune system ^32^ and our findings illustrate how stratification of patients by airway microbiome may have theragnostic potential.

IL‐13 production in the context of an abundance of potentially pathogenic bacteria may be serving a unique function in comparison to IL‐13 produced as part of the allergic cascade. IL‐13 plays an important role in epithelial repair via heparin binding epidermal growth factor (HB‐EGF) ^26^ and IL‐13 expression has been shown to closely mirror epithelial HBEGF expression in a sub cluster of severe asthma patients with evidence of neutrophilic inflammation from the U‐BIOPRED cohort.^27^ The generation of IL‐13 in the presence of bacteria and neutrophilic inflammation may be an important homeostatic mechanism to limit epithelial damage and enhance the wound repair responses.

Even though we have replicated our findings, there are limitations to our study. We have used the separation of polarized groups to understand the biological relationships. This is justified by the exploratory nature of our study of variables not non‐normally distributed within the study population ^33^ but means that linear relationships between IL‐13, BAL neutrophils and bacterial colonization cannot be assumed. Formal testing of this did, however, identify a significant relationship between potentially pathogenic bacterial abundance and both BAL neutrophils and BAL IL‐13. Concomitant bronchiectasis is a factor that could influence the airway microbiome. None of the severe asthma patients had bronchiectasis as a significant clinical diagnosis and whilst thoracic CT scan imaging is available on many, not all had strict confirmation or exclusion of bronchiectasis. The severe asthmatics have a history of recurrent severe exacerbations and to limit the impact of this, no bronchoscopies were undertaken within 8 weeks of an exacerbation. Nevertheless, this will be a population that has exposure to oral steroid bursts and antibiotic therapy.

This steroid unresponsive source of IL13 is associated with BAL neutrophilia and bacterial dysbiosis, highlighting the importance of a greater understanding of the innate and adaptive immune responses in the peripheral airways of severe asthma and how they relate to clinical disease expression. Better appreciation and understanding of the different endotypic mechanisms leading to IL13 production is critical in being able to offer targeted therapies.

## CONFLICT OF INTERESTS

Dr. Azim has nothing to disclose. Dr. Green has nothing to disclose. Dr. Lau has nothing to disclose. Dr. Rupani has nothing to disclose. Dr. Jayasekera has nothing to disclose. Dr. Bruce has nothing to disclose. Prof. Howarth reports employment by and has shares in GSK.

## AUTHOR CONTRIBUTION

AA performed the statistical analysis, drafted the manuscript and designed the figures. BG, HR and NJ performed the clinical characterization and biological sampling of the participants. LL performed electrochemiluminescence immunoassays and KB performed the terminal restriction fragment length polymorphism. PH conceived and oversaw overall direction and planning of the study. All authors provided critical feedback and helped shape the research, analysis and manuscript.

## Supporting information

Supplementary Material

Supplementary Material
