# Supplementary material for: Peripheral airways type 2 inflammation, neutrophilia and microbial dysbiosis in severe asthma
Source: Allergy. 2021 Jan 26;76(7):2070–8. doi: 10.1111/all.14732 (PMC8629111; doi:10.1111/all.14732)
Supplement: Supplementary file 1 — Supplementary Material [file ALL-76-2070-s001.docx]

# SUPPLEMENTARY MATERIAL

|  | Healthy Volunteers  *(45)* | Mild Asthma  *(80)* | Severe Asthma  *(78)* | P value |
| --- | --- | --- | --- | --- |
| BAL IL-13 | 3.02 (2.23) | 2.87 (1.66) | 4.60 (3.74) | <0.001 |
| P value vs Healthy | */* | *ns* | *p<0.001* |  |
| P value vs Mild | *ns* | */* | *p<0.001* |  |
| BAL IL-4 | 0.18 (0.16) | 0.12 (0.10) | 0.20 (0.32) | <0.001 |
| P value vs Healthy | */* | *p=0.025* | *ns* |  |
| P value vs Mild | *p=0.025* | */* | *p<0.001* |  |
| BAL IL-5 | 0.39 (0.29) | 0.48 (0.38) | 0.46 (0.58) | ns |
| P value vs Healthy | */* | *ns* | *ns* |  |
| P value vs Mild | *ns* | */* | *ns* |  |

***TABLE E1*** *– Bronchoalveolar lavage concentrations of type 2 cytokines in healthy volunteers, mild asthma and severe asthma. Between group comparisons by Kruskall Wallis test with pairwise comparisons corrected for multiple comparisons by Dunn’s Method. IL = interleukin.*

|  | Healthy Volunteers  *(45)* | Mild Asthma  *(80)* | Severe Asthma  Low IL13  *(25)* | Severe Asthma High IL13  *(25)* | P value |
| --- | --- | --- | --- | --- | --- |
| BAL Eosinophils (%) | 0.25 (0.50) | 1.00 (2.20) | 0.25 (0.50) | 0.75 (2.13) | p<0.001 |
| BAL Eosinophils/ml | 9 x10^5^  (0.00057) | 138 x10^5^ (0.00465) | 50 x10^5^  (0.00190) | 78 x10^5^  (0.00316) | p<0.001 |
| BAL Neutrophils (%) | 2.30 (3.65) | 2.00 (2.44) | 3.30 (5.65) | 23.00 (33.15) | p<0.001 |
| BAL Neutrophils/ml | 236 x10^5^  (0.00613) | 365 x10^5^  (0.00694) | 566 x10^5^  (0.00963) | 2218 x10^5^  (0.04999) | p<0.001 |

***TABLE E2*** *– Bronchoalveolar lavage inflammatory cell counts in healthy volunteers, mild asthma and severe asthma patients from the top and bottom tertile of patients ranked by Interleukin 13 concentrations. Between group comparisons by Kruskal Wallis test. BAL = bronchoalveolar lavage*

|  | Healthy Volunteers  *(45)* | Mild Asthma    *(80)* | Severe Eosinophilic  *(9)* | Severe Neutrophilic  *(26)* | Severe  Mixed  *(8)* | Severe  Pauci  *(35)* |
| --- | --- | --- | --- | --- | --- | --- |
| BAL IL-4 | 0.18 (0.16) | 0.13 (0.10) | 0.31 (0.24) | 0.24 (0.67) | 0.50 (2.53) | 0.18 (0.13) |
| p value vs Mild Asthma | *p=0.042* | */* | *ns* | *p<0.001* | *p=0.003* | *p=0.043* |
| BAL IL-5 | 0.39 (0.29) | 0.48 (0.38) | 1.44 (2.53) | 0.52 (0.70) | 1.06 (4.08) | 0.35 (0.23) |
| p value vs Mild Asthma | *ns* | */* | *ns* | *ns* | *ns* | *ns* |
| BAL IL-13 | 3.02 (2.23) | 2.87 (1.66) | 4.80 (2.15) | 6.16 (5.39) | 8.77 (13.85) | 3.22 (2.43) |
| p value vs Mild Asthma | *ns* | */* | *ns* | *p<0.001* | *p<0.001* | *ns* |

***TABLE E3*** *– Type 2 Cytokine concentrations in healthy volunteers, mild asthma and severe asthma patients divided into inflammatory phenotypes. Between group comparisons by Kruskal Wallis test with pairwise comparisons corrected for multiple comparisons by Dunn’s Method. BAL = bronchoalveolar lavage, IL = interleukin.*

#

# ***FIGURE E1*** *– Bronchoalveolar Inflammatory Cell Counts, (A Eosinophils, B Neutrophils) across Healthy volunteers, Mild Asthma, Severe Asthma with Low IL-13 and Severe Asthma with High IL-13 in Replicate Cohort*
