# Supplementary figures and images for: Peripheral airways type 2 inflammation, neutrophilia and microbial dysbiosis in severe asthma
Source: Allergy. 2021 Jan 26;76(7):2070–8. doi: 10.1111/all.14732 (PMC8629111; doi:10.1111/all.14732)

**A**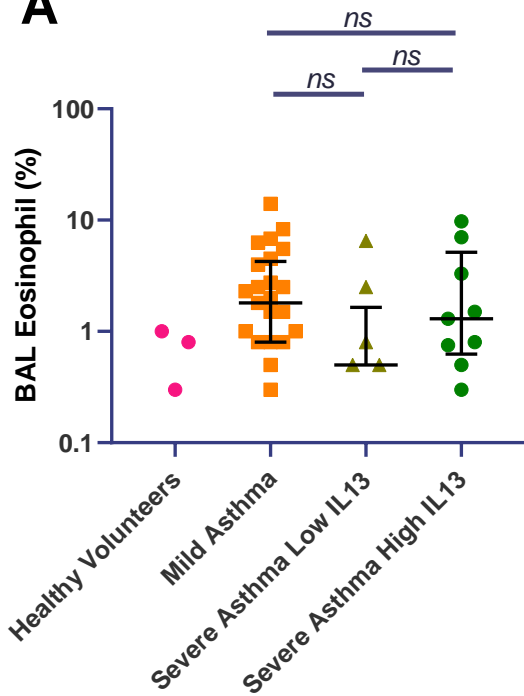**B**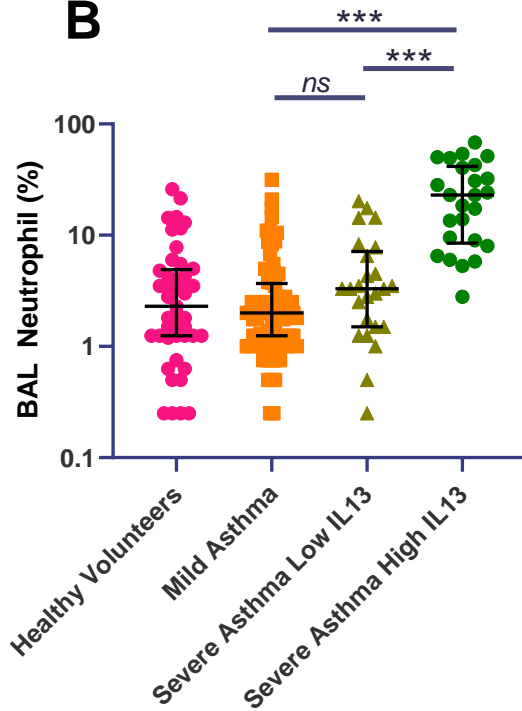

Supplement: Supplementary file 2 — Supplementary Material [file ALL-76-2070-s002.pdf]
